# Psychological Well-Being, Knowledge Management Behavior and Performance: The Moderating Role of Leader-Member Exchange

**DOI:** 10.3389/fpsyg.2021.566516

**Published:** 2021-05-11

**Authors:** Cheol Young Kim

**Affiliations:** School of Business Administration, Myongji University, Seoul, South Korea

**Keywords:** psychological well-being, knowledge-sharing behavior, knowledge-hiding behavior, knowledge-manipulating behavior, job performance, leader-member exchange

## Abstract

Knowledge is considered an essential resource and key to competitiveness. The behavior of sharing knowledge is an essential activity for the prosperity of the organization. For individuals, however, sharing knowledge can present a dilemma by giving up the exclusive right to certain knowledge that they own. This study identifies the psychological well-being as a leading factor in facilitating knowledge-sharing in dilemma situations. The author classified knowledge management behavior into sharing, hiding, and manipulating behavior, and studied them as mediators linking psychological well-being and performance. And to check the influence of the quality of the exchange relationship, leader-member exchange was used as a moderator. For the empirical analysis, 333 members from 12 organizations were surveyed by using different sources and times. Hierarchical regression analysis and bootstrapping analysis were conducted for verification of hypothesis. Results demonstrated that the psychological well-being influence directly on knowledge-sharing, -hiding, and -manipulating behaviors and indirectly on performance. In the multi-mediation test, only knowledge-sharing behavior mediated the relationship between psychological well-being and performance. And the moderating effect of leader-member exchange was significant only in the relationship between psychological well-being and knowledge-sharing behavior. This study contributes to the performance, knowledge management and positive psychology research fields, and suggests practical implications.

## Introduction

Since the early Hawthorne studies in business literature, the causes of high productivity and the performance of happy employees have been widely known (Roethlisberger and Dickson, 1939). The happier the members, the more they dedicate themselves to their task, produce creative ideas, and participate in organizational citizenship behaviors. Traditionally, this causal relationship has been accepted because of the mutually beneficial relationship in which a person exchanges favor with favor (Eagly and Chaiken, 1993; Jena et al., 2018). However, this causal relationship, unlike the direction the theory points out, has not been demonstrated. Until the 1950s, the relationship was not proven in empirical analysis (Brayfield and Crockett, 1955), and until the 1990s empirical analyses of the significance of the relationship were mixed (Shore and Martin, 1989; Keaveney and Nelson, 1993). There are two reasons why this relationship is difficult to demonstrate despite the seemingly clear theoretical direction. First, the definition of happiness varies by study. Happiness has been defined as satisfaction with an organization or job, a positive emotion, or mental health. Because the concept of happiness is so wide and each scholar uses different definitions and measures, the study direction varies according to the research and the empirical results differ. Second, because happiness is the overall psychological state of the members, it is conceptually distant from the performance that results from behavior. Various behavioral and situational factors, including leadership, are involved in job performance, and the causal relationship between happiness and performance is distorted in this process. Therefore, this paper aims to investigate the process concerning member happiness and performance by using psychological well-being concepts to define happiness, identifying processes that lead to performance, and analyzing the impact of leadership.

Knowledge is a key resource for the performance of individuals and organizations (Lin, 2007), essential for generating new information, correcting errors, analyzing tasks, and creating value (Ohlsson, 2011). It is also a key element in innovation, which is essential for organizations in modern society (Lin, 2007). Unlike other material resources for organizational performance, knowledge does not diminish in the process of sharing with others; rather, the possibility of new knowledge being created and receiving knowledge from others increases. From an organizational perspective, knowledge-sharing is an act of expanding the resources of the entire organization and is a major way of improving organizational performance (Darroch, 2005). Moreover, in the current business environment where digitization, homework, and online activities are increasingly spreading, the importance of knowledge sharing among employees is increasing (Chumg et al., 2015; Ordieres-Meré et al., 2020). However, from each member’s point of view knowledge-sharing is not an absolute good. Rather, it is a dilemma. A knowledgeable individual is highly-valued as a human resource and receives greater rewards. Sharing knowledge is the act of giving up one’s exclusive position in that unique knowledge area. Thus, an organization member has an incentive to hide knowledge without sharing it. The motivations to share or hide knowledge are often studied as a conflict between individual interests and group interests (Kimmerle et al., 2011). Members who dedicate themselves to the organization and internalize the goals of the organization are active in knowledge-sharing, whereas members who strive to preserve position or status will hide their knowledge and seek profits from knowledge-exchange by overstating their knowledge and underestimating others’ knowledge.

Which members of the organization are more actively sharing knowledge without hiding or manipulating it? Despite existing studies of knowledge management behavior, there is a research gap on the factors that solve this knowledge sharing dilemma. This study was conducted with the aim of solving the knowledge sharing dilemma in two psychological aspects. First, the higher the psychological well-being, the easier it will be to overcome the dilemma. Second, the higher the quality of the exchange relationship with the leader, the more actively participate in the exchange relationship and more easily overcome the dilemma. In other words, this paper aims to confirm that employees with high psychological well-being and leader-member exchange are more active in sharing knowledge and will eventually achieve higher performance.

## Theory and Hypothesis

### Knowledge-Management Behavior

Knowledge is essential to organizational competitiveness and growth (Grant, 2002). It is a key resource for value-added processes and a major catalyst for innovation. The organization seeks to hire and acquire members as important human resources for acquiring knowledge, and knowledgeable individuals are managed as major human resources in the organization and enjoy relatively high status and rewards (Brown and Duguid, 1991). The knowledge formed by the individual members is propagated in the organization through knowledge-sharing behaviors, and the knowledge that is sufficiently shared remains in the organization and becomes social capital (organizational knowledge). According to previous research, organizations with rich knowledge can reduce production costs, develop new products quickly, have high innovative capabilities, and generate high-quality products and services (e.g., Cummings, 2004; Collins and Smith, 2006). Therefore, organizations seek to promote knowledge-sharing behavior in several ways.

Contrary to the wishes of the organization, however, its members have two different motives for distributing their knowledge and employ three behavioral strategies according to each combination. The first motivation is to actively distribute knowledge. This motivation is expressed based on two rewards. The knowledge provided to others is directly returned to respect and recognition and indirectly rewarded with valuable knowledge. By sharing knowledge externally, they reveal their knowledge to the organization, receive verification of value, and receive monetary compensation, promotion, and expansion of authority. If this incentive is strong, members become active in knowledge-sharing behavior. Accordingly, knowledge-sharing behaviors are defined as the provision of work information or know-how to help others (Wang and Noe, 2010).

The second motivation is the desire to reject knowledge circulation and monopolize knowledge. Knowledge is a valuable intangible resource that contributes to the effectiveness of an individual or organization and contributes to creating new knowledge. If competitors have access to the knowledge of an organization, the scarcity of knowledge decreases and undermines the organization’s relative position by promoting the performance of its competitors. If a particular member monopolizes certain knowledge, others must obtain permission from him or her to gain access to the knowledge (Wang et al., 2019). The more valuable the knowledge, the more valuable that member’s status will be. Knowledge-hiding behavior is thus defined as the exclusive use of knowledge requested by others (Connelly et al., 2012). Members with strong first motivations will actively engage in knowledge-sharing behavior, and members with strong second motivations will engage in knowledge-hiding behavior. These two motivations may occur simultaneously based on the circumstances. A one-sided deal cannot occur. Members must share their knowledge for others to receive it. Consequently, members have an incentive to overestimate the validity of their knowledge and to underestimate the importance of others’ knowledge to promote profit in the transaction–they balance the incentive to share knowledge with the incentive to monopolize it through the benefit of knowledge transactions. This is defined as knowledge-manipulation behavior (Bettis-Outland, 1999), one of three types of knowledge-management behaviors, the others being knowledge-sharing and knowledge-hiding (Rhee and Choi, 2017).

The two motivations that cause the three knowledge-management behaviors vary by context. First, the culture, institutions, and atmosphere of the organization affect the two motives. Trust in members was studied to mitigate the adverse effects of knowledge-sharing and increase the frequency of communication, thus boosting motivation for sharing knowledge (Kankanhalli et al., 2005). Organizations with a knowledge-management system remove the physical barriers to facilitating knowledge-sharing (Chiu et al., 2006; Collins and Smith, 2006). Members in an atmosphere that rewards or actively encourages knowledge-sharing are also motivated to share knowledge (Bock et al., 2005), and knowledge-sharing is active in organizations with a learning culture (Taylor and Wright, 2004). If an organization does not have a compensation system for knowledge, it will stimulate motivation for monopoly knowledge (Yao et al., 2007). Conversely, the use of knowledge-management systems is stimulated in organizations with monetary rewards such as promotions and salary increases (Kankanhalli et al., 2005). In Korean corporate samples, collective pay systems stimulate knowledge-sharing (Kim and Lee, 2006). However, research results are mixed regarding monetary compensation. Several studies suggest that external rewards, such as monetary rewards, weaken intrinsic motivations and promote knowledge-hiding: intrinsic motivation is more important in knowledge-sharing behavior (Bock and Kim, 2002; Kwok and Gao, 2005). In addition to these organizational and external factors, factors within individuals also affect the two knowledge-management motivations. The exchange ideology affects the exchange of knowledge (Lin, 2007). Among personality factors, the more openness to experience, the greater the knowledge-sharing motivation (Cabrera et al., 2006). The more a member is familiar with information technology (IT), the more actively he or she uses the knowledge-management system (Jarvenpaa and Staples, 2000). The more educated and knowledgeable the members, the more they pursue growth through knowledge exchange (Constant et al., 1994).

### Psychological Well-Being, Knowledge-Management Behaviors, and Performance

The psychological well-being of organizational members is a field of happiness research that has been studied for thousands of years since Aristotle–at a macroscopic level–and a central theme of psychological research of members in business administration since the Hawthorne study–at a microscopic level. The definition of psychological well-being differs slightly among scholars.

This study deals with the evaluation of the members themselves in organizational situations and the resulting behavioral changes, thus realizing and completing each member’s potential in job situations base on the definition of Ryff (1989), which is widely accepted in this respect. Psychological well-being is high for those who learn and identify new ways of solving problems in the process of achieving their goals, performing their jobs successfully, and solving problems. First, members with high psychological well-being have a strong belief in themselves. Through self-efficacy, they enjoy challenges, are engaged in work, and strive to reach goals (Bandura, 1997; Garg and Rastogi, 2009). Because they recognize themselves as members of the organization and feel happy, they internalize the organization’s goals more easily. They align their goals with the organization’s goals and share their knowledge for organizational success. Because of their high self-evaluation and not judging themselves by others, their motivation to overestimate and manipulate their information to gain profits in knowledge-sharing is weak.

Second, members with high psychological well-being are optimistic about the consequences of knowledge-sharing. The relationship between cost and reward in social exchange is the most widely-studied topic in social exchange relations, including knowledge-sharing (Wang and Noe, 2010). People with high psychological well-being are optimistic about the exchange. A strong optimist perceives the outcome of events in a positive direction and reduces the impact of negative events. They set higher goals with higher quality norms of reciprocity, quickly eliminating frustration (Luthans et al., 2004). Therefore, they consider the rewards of knowledge-sharing valuable and the cost small, and they have a strong motivation to reward knowledge-sharing from others. They will actively participate in knowledge-sharing and will not hide or manipulate knowledge.

*Hypothesis 1. Psychological well-being and knowledge-sharing behaviors are positively related.*

*Hypothesis 2. Psychological well-being and knowledge-hiding behaviors are negatively related.*

*Hypothesis 3. Psychological well-being and knowledge-manipulating behaviors are negatively related.*

Members who actively use their knowledge for the goals of the organization are likely to perform well. From an intrinsic perspective, they have a positive evaluation of themselves and identify resources to dedicate themselves toward reaching their goals. Instead of hiding or manipulating knowledge, they actively use their knowledge without considering others. Knowledge-sharing stimulates peer knowledge-sharing, increases the total amount of knowledge across the organization, and contributes to organizational performance (Collins and Smith, 2006). Members participating in knowledge-sharing behavior are likely to receive valuable knowledge from their peers. According to the signaling effect, exposing knowledge also increases evaluation and status in the organization and thus increases the likelihood of receiving necessary resources (Cropanzano and Mitchell, 2005). Increasing organizational knowledge, in turn, leads to an increase in personal knowledge, and tacit knowledge is revealed and clarified. Consequently, performance increases.

Conversely, knowledge-hiding behavior makes it difficult for members to reveal their knowledge or encourage others or leaders to devalue the knowledge. Their status in the organization is lowered and, in particular, separated from the knowledge-sharing network (Rhee and Choi, 2017). Knowledge is not distributed in social exchange relationships with colleagues, which makes it difficult to acquire the resources necessary for a member to increase performance. Inherently, they have a “free ride” in organizational knowledge, which lowers their self-evaluation and makes them feel unfair in their relationships with others. Consequently, performance decreases.

Knowledge manipulation behaviors are strategic actions to share knowledge while seeking benefits in the process. Members who engage in these behaviors exaggerate their knowledge, underestimate the risks, and emphasize the benefits. They cannot dedicate themselves to the process and are less likely to contribute to the goals of the organization. The distortion of knowledge they cause inevitably leads to degradation of performance.

*Hypothesis 4. Knowledge-sharing behavior and performance are positively related.*
*Hypothesis 5. Knowledge-hiding behavior and performance are negatively related.*
*Hypothesis 6. Knowledge-manipulating behavior and performance are negatively related.*
*Hypothesis 7. The relationship between psychological well-being and performance is mediated by* (*a*) *knowledge-sharing behavior*, (*b*) *knowledge-hiding behavior, and* (*c*) *knowledge-manipulating behavior.*

### The Moderating Effect of Leader-Member Exchange

The leader is the symbol of the organization and the source of resources (Yukl, 1989). Organizational leadership has a direct impact on the knowledge-sharing motivation of members. The higher management evaluates the knowledge, the greater the knowledge-sharing among the members (Lee et al., 2006). Perceived organizational support and empowering leadership increase the effectiveness of knowledge-sharing (Cabrera et al., 2006; Srivastava et al., 2006). Transformational leadership encourages members to dedicate themselves to the organization, internalize organizational goals, and share knowledge for their own growth (Bryant, 2003). Therefore, the relationship with the leader is an important situational factor in the behavior of members.

Leader-member exchange (LMX) is part of social exchange theory (Blau, 1964). Leaders do not form a homogeneous exchange relationship with their members. They form a reciprocal exchange relationship by in-grouping some of the members, and some of the members are out-grouped to create an unequal exchange relationship (Dansereau et al., 1975). Members form their own standards of behavior according to the quality of this exchange relationship. Members with high LMX form a reciprocal exchange relationship with the leader. They communicate frequently with leaders and receive resources. Consequently, satisfaction increases for both leaders and subordinates (Liden and Graen, 1980). Members are actively immersed in the organization based on their trust in the leader. If members with high psychological well-being have a high quality of relationships with their leaders, they are optimistic about the outcome of the exchange and confident that they will be highly regarded by the organization through knowledge-sharing. They think the leader will acknowledge their contribution, and they take more risks in the exchange relationship based on a belief in their leader. They also regard knowledge-hiding and knowledge-manipulating behaviors as betrayals of the leader, making an effort to reduce these actions. Even though they have low psychological well-being, they can actively participate in knowledge-sharing behavior based on their trusting relationships with leaders. However, members with low LMX tend to do the opposite. Members who think they are an outsider finds it difficult to take the risk of sharing knowledge, even if their psychological well-being is high. Because they are not satisfied with their exchange relationship with the leader, it is difficult to engage in knowledge-exchange relationships in the organization. They seek to protect themselves by hiding their knowledge, or they exaggerate and seek the benefit of the knowledge-exchange process, rather than risk lowering their position by exposing their knowledge.

*Hypothesis 8a. The relationship between psychological well-being and knowledge-sharing behavior, are moderated by LMX. such that the relationship is stronger when LMX is high rather than low.*
*Hypothesis 8b. The relationship between psychological well-being and knowledge-hiding behavior, are moderated by LMX. such that the relationship is weaker when LMX is high rather than low.*
*Hypothesis 8c. The relationship between psychological well-being and knowledge-manipulating behavior are moderated by LMX. such that the relationship is weaker when LMX is high rather than low.*

## Materials and Methods

### Data Collection and Study Subjects

Data for the empirical analysis was collected through a pencil-and-paper survey from organizations in South Korea. Twelve companies from financial, manufacturing, distribution, IT, construction, and public institutions participated in the study to secure the generalizability and external viability of results without being biased to specific industries. Participants were informed that a researcher at Seoul National University would conduct research on working conditions and employee happiness. To reduce common method variance. I distributed surveys to 450 leader–member dyads at two points in time. At Time 1 members rated their psychological well-being, perceived LMX, and demographic data. At Time 2 (1 month later) members rated their knowledge management behaviors, and leaders rated subordinates’ in-role performance. All participants were assigned random numbers to match the first and second set of questionnaires with the answers of the leaders and members. The questionnaires were distributed to leader-member dyads. 382 copies were received from the first questionnaire and 366 from the second questionnaire. After matching the questionnaires, the remaining 333 samples were used for hypothesis testing: 70.3% are male and 29.7% female, 72.1% are married, 51.7% are college graduates, and 40.2% work in R&D jobs.

### Measures

All measures were translated into Korean by two organizational behavior professors. A seven-point Likert scale ranging from 1 (strongly disagree) to 7 (strongly agree) was used to assess all measures.

#### Psychological Well-Being

Psychological well-being, an independent variable, was measured using the 18-item scale proposed by Ryff and Keyes (1995). One example includes “For me, life has been a continuous process of learning, changing, and growth.” It was collected in the first survey in the form of a self-report. The reliability (Cronbach’s α) was 0.85. This measure was previously used in Joo et al. (2016), and the reliability was 0.91.

#### Job Performance

Job performance, a dependent variable, was measured using the seven-item job performance measuring tool proposed by Williams and Anderson (1991) and used in Bernerth et al. (2012, α = 0.87). In the second survey, the supervisor responded about the subordinate. Examples include “He/She adequately completes assigned duties” and “He/she engages in activities that will directly affect his/her performance evaluation.” The Cronbach’s α was 0.91.

#### Knowledge-Sharing Behavior

All three knowledge-management behaviors were measured using the four-item scales proposed by and used in Rhee and Choi (2017). The reliability of knowledge-sharing behavior in that study was 0.81. Knowledge-sharing behavior was answered by subordinates in the second survey. Examples include “I looked into the request to make sure my answers were accurate” and, “I told my coworker exactly what he/she needed to know.” The Cronbach’s α was 0.91.

#### Knowledge-Hiding Behavior

Knowledge-hiding behavior was measured by subordinates responding to the second survey. Examples include “I pretended that I did not know the information” and, “I said that I did not know even though I did.” The Cronbach’s α was 0.88, slightly higher than the previous study’s 0.86 (Rhee and Choi, 2017).

#### Knowledge-Manipulating Behavior

Knowledge-manipulating behavior was measured by subordinates responding to the second survey. Examples include “I padded my knowledge to make it greater than it actually is” and “I omitted potential problems inherited from my knowledge.” The Cronbach’s α was 0.81, which was the same as the previous study (Rhee and Choi, 2017).

#### Leader-Member Exchange

Leader-member exchange, which is a moderating variable, was measured in the first questionnaire by subordinates responding using the seven-item LMX scale proposed by Graen and Uhl-Bien (1995) and used in Chaurasia and Shukla (2013, α = 0.85). Examples include “How well does your leader understand your job problems and needs?” and “Regardless of how much formal authority he/she has built into his/her position, what are the chances that your leader would use his/her power to help you solve problems in your work?.” The Cronbach’s α was 0.78.

### Data Analysis

Using R 3.5.2 software, I tested hypotheses 1 through six using multiple regression analysis. I controlled for gender and age for all models at step 1 and entered the main effect at step 2. The main effect should explain a significant amount of the variance of the dependent variable for supporting hypotheses. And I tested mediation hypothesis 7 using PROCESS macro developed by Preacher and Hayes (2004). The 95% bias-corrected confidence interval should not be containing zero for supporting hypothesis. Lastly, I tested the moderation hypothesis 8 using multiple regression analysis. For supporting the moderation effect, the interaction term between psychological well-being and LMX should explain a statistically significant amount of the variance of the dependent variable. In addition, the effect size of psychological well-being should increase as LMX increases in a sensitivity analysis.

## Results

To verify the discriminant validity of the translated measures, the factor structures of each variable measured at the individual level were checked through confirmatory factor analysis (CFA). The hypothesized six-factor model yielded an acceptable fit to the data (*χ*^2^ = 1,344.72, *p* < 0.01, comparative fit index = 0.92, Tucker-Lewis index = 0.93, root mean square error of approximation = 0.054, Hu and Bentler, 1999).

### Hypothesis Testing

Table 1 reports the means, standard deviations, and correlations for all variables. Reliability is high, at greater than.78 for all variables. The correlation between knowledge-sharing and knowledge-hiding behavior is −0.24. The relationship between LMX and performance is significant. As a result of correlation analysis, most variables have the same direction as the hypotheses.

**TABLE 1:** Means, standard deviations, and correlations for all variables.

	**Variables**	**Mean**	**SD**	**1**	**2**	**3**	**4**	**5**	**6**	**7**	**8**
1	Gender	0.28	0.45	1.00							
2	Age	37.21	6.78	−0.19**	1.00						
3	PWB	4.31	0.72	−0.07	0.03	(0.85)					
4	KSB	4.84	1.18	−0.14**	−0.06	0.40**	(0.91)				
5	KHB	4.03	1.06	0.04	0.00	−0.30**	−0.24**	(0.88)			
6	KMB	3.64	1.14	0.08	−0.01	−0.30**	−0.42**	0.47**	(0.81)		
7	Performance	4.60	1.16	−0.12*	−0.06	0.12*	0.46**	−0.13*	−0.27**	(0.80)	
8	LMX	4.59	1.06	−0.13*	−0.04	−0.14*	0.13*	0.18**	0.00	0.40**	(0.78)

PWB, psychological well-being; KSB, knowledge sharing behavior; KHB, knowledge hiding behavior; KMB, knowledge manipulating behavior; LMX, leader-member exchange. *p < 0.05; **p < 0.01.

A hierarchical regression analysis was performed for testing hypothesis. Gender and age were controlled for all models. The analysis results of the three knowledge-management behaviors (knowledge-sharing, knowledge-hiding, and knowledge-manipulating) are displayed in Table 2.

**TABLE 2:** Results of hierarchical linear regression analysis for hypothesis 1, 2, and 3.

**Variables**	**Knowledge sharing behavior**				**Knowledge hiding behavior**				**Knowledge manipulating behavior**			
	**Model 1**		**Model 2**		**Model 3**		**Model 4**		**Model 5**		**Model 6**	
	***b***	**s.e.**	***b***	**s.e.**	***b***	**s.e.**	***b***	**s.e.**	***b***	**s.e.**	***b***	**s.e.**
Constant	5.56**	0.37	2.82**	0.49	3.95**	0.34	5.83**	0.46	3.59**	0.37	5.61**	0.50
Gender	−0.41**	0.15	−0.34*	0.13	0.09	0.13	0.04	0.13	0.20	0.14	0.15	0.14
Age	−0.02	0.01	−0.02	0.01	0.01	0.01	0.01	0.01	0.01	0.01	0.01	0.01
PWB			0.64**	0.08			−0.44**	0.08			−0.47**	0.08
*R*^2^	0.03		0.18		0.01		0.09		0.01		0.09	
△*R*^2^			0.15				0.08				0.08	
Adj. *R*^2^	0.02		0.17		0.01		0.08		0.01		0.09	
*F*-value	4.70*		23.80**		0.25		10.85**		1.01		11.35**	

PWB, psychological well-being. *n* = 333; *p < 0.05; **p < 0.01.

The regression coefficient of psychological well-being is positive on knowledge-sharing behavior (Model 2, *b* = 0.64, *p* < 0.01) and negative on knowledge-hiding behavior (Model 4, *b* = −0.44, *p* < 0.01) and knowledge-manipulating behavior (Model 6, *b* = −0.47, *p* < 0.01). In the three models, the regression coefficients for psychological well-being are statistically significant, supporting hypotheses 1, 2, and 3. The analysis results of the three knowledge-management behaviors (knowledge-sharing, knowledge-hiding, and knowledge-manipulating) on job performance are displayed in Table 3.

**TABLE 3:** Results of hierarchical linear regression analysis for hypothesis 4, 5, and 6.

**Variables**	**Job performance**							
	**Model 7**		**Model 8**		**Model 9**		**Model 10**	
	***b***	**s.e.**	***b***	**s.e.**	***b***	**s.e.**	***b***	**s.e.**
Constant	5.26**	0.37	2.84**	0.43	5.82**	0.43	6.23**	0.40
Gender	−0.36*	0.14	−0.18	0.13	−0.35*	0.14	−0.31*	0.14
Age	−0.02	0.01	−0.01	0.01	−0.02	0.01	−0.02	0.01
Knowledge Sharing Behavior			0.44**	0.05				
Knowledge Hiding Behavior					−0.14*	0.06		
Knowledge Manipulating Behavior							−0.27**	0.05
*R*^2^	0.02		0.21		0.04		0.09	
△*R*^2^			0.19		0.02		0.07	
Adj. *R*^2^	0.02		0.21		0.03		0.09	
*F*-value	3.79*		29.90**		4.48**		11.22**	

*n* = 333; *p < 0.05; **p < 0.01.

The results show that knowledge-sharing behavior (Model 8, *b* = 0.44, *p* < 0.01) is significantly positive and knowledge-hiding behavior (Model 9, *b* = −0.14, *p* < 0.05) and knowledge-manipulating behavior (Model 10, *b* = −0.27, *p* < 0.01) are significantly negative on job performance. Hypothesis 4, 5, and 6 is supported.

To verify the mediation effects of the three knowledge-management behaviors (knowledge-sharing, knowledge-hiding, and knowledge-manipulating), the indirect effects were analyzed by creating a multi-mediation model with three behaviors simultaneously. The results are presented in Table 4.

**TABLE 4:** The bootstrapping results of mediation.

**Predictor: psychological well-being Outcome: job performance**			**Bias corrected bootstrap 95% confidence interval**	

**Mediator:**	**Indirect effect**	**Boot SE**	**Lower**	**Upper**
Knowledge sharing behavior	0.233	0.047	0.169	0.389
Knowledge hiding behavior	−0.001	0.029	−0.063	0.056
Knowledge manipulating behavior	0.055	0.034	−0.004	0.131

*Bootstrapping based on *n* = 20,000 subsamples.*

From the bootstrapping analysis, only indirect effects through knowledge-sharing behavior are significant in the multi-mediation model, and the path through the knowledge-hiding or knowledge manager is not significant. Thus, hypothesis 7 is partially supported. The results of the moderating effect analysis are presented inTables 5, 6.

**TABLE 5:** Results of moderating analysis for hypothesis 8.

**Variables**	**Knowledge sharing behavior**				**Knowledge hiding behavior**				**Knowledge manipulating behavior**			
	**Model 11**		**Model 12**		**Model 13**		**Model 14**		**Model 15**		**Model 16**	
	***b***	**s.e.**	***b***	**s.e.**	***B***	**s.e.**	***b***	**s.e.**	***b***	**s.e.**	***B***	**s.e.**
Constant	1.69**	0.59	5.96**	1.47	4.97**	0.56	7.06**	1.41	5.84**	0.61	5.76**	1.54
Gender	−0.27*	0.13	−0.25	0.13	0.09	0.13	0.10	0.13	0.13	0.14	0.13	0.14
Age	−0.02	0.01	−0.01	0.01	0.01	0.01	0.01	0.01	0.01	0.01	0.01	0.01
PWB	0.68**	0.08	−0.26	0.31	−0.41**	0.08	−0.86**	0.29	−0.48**	0.08	−0.46	0.32
LMX	0.19**	0.06	−0.84*	0.33	0.14**	0.05	−0.36	0.31	−0.04	0.06	−0.02	0.34
PWB*LMX			0.22**	0.07			0.11	0.07			−0.01	0.07
*R*^2^	0.21		0.23		0.11		0.12		0.10		0.10	
△*R*^2^			0.02				0.01				0.00	
Adj. *R*^2^	0.17		0.24		0.10		0.11		0.08		0.08	
*F*-value	21.22**		19.47**		10.12**		8.65**		8.61**		6.87**	

PWB, psychological well-being; LMX, leader-member exchange. *n* = 333; *p < 0.05; **p < 0.01.

**TABLE 6:** Sensitivity analysis of results on the moderating effect of the leader-member exchange.

**Predictor: psychological well-being Outcome: knowledge sharing behavior**					**Bias corrected bootstrap 95% confidence interval**	

**Moderater**	**Effect size**	**SE**	***T***	***p***	**Lower**	**Upper**
Low LMX	0.526	0.094	5.62	<0.01	0.341	0.710
Mid LMX	0.761	0.085	8.98	<0.01	0.594	0.928
High LMX	0.997	0.129	7.73	<0.01	0.743	1.25

*LMX, leader-member exchange. Bootstrapping based on *n* = 20,000 subsamples.*

Although the regression coefficients of the interaction term between psychological well-being and LMX is significant for knowledge-sharing behavior (Model 12, *b* = 0.22, *p* < 0.01), regression coefficients of the knowledge-hiding and knowledge-manipulating behaviors are not statistically significant.

The effect size of psychological well-being on knowledge-sharing when the LMX relationship is low is 0.526 and increases to 0.761 and 0.997 as the LMX relationship increases. Thus, hypothesis 8a is supported. Hypotheses 8b and 8c, which have insignificant regression coefficients, are rejected.

## Discussion

This study classified knowledge-management behavior into three actions: knowledge-sharing, knowledge-hiding, and knowledge-manipulating. This study hypothesized a change in performance based on psychological well-being through these knowledge-management behaviors. The LMX relationship was regarded as a situational factor. From the empirical analysis, the direct effects of psychological well-being on knowledge-sharing, knowledge-hiding, and knowledge-manipulating behavior are all significant, and all of these behaviors are related to performance. The multi-mediation bootstrapping analysis, which confirmed the mechanism for the relationship between psychological well-being and performance, found that knowledge-sharing is the most important route because its path is the only significant path. In previous studies, the mediating effect of knowledge management behavior has been discussed, but the difference between the three types of knowledge management behavior has not been studied (e.g., Rhee and Choi, 2017). The results of this study show differences in the relative importance of knowledge management behaviors. The moderating effect of LMX is significant only for the relationship between psychological well-being and knowledge-sharing behavior. The role of LMX as the primary route for knowledge-sharing behavior is supported. This is consistent with previous studies that LMX acts as an important context variable in the relationship between knowledge donating and knowledge collecting (Dysvik et al., 2015).

### Contributions and Implications

This paper has several academic contributions. First, the relationship between psychological well-being and performance was less supported by empirical analysis than by theory (Carolan et al., 2017). This paper identifies the causal relationship between the two concepts through three types of knowledge-management behaviors. The knowledge-sharing, hiding, and manipulating behaviors are determined by the degree of psychological well-being, and these behaviors determine job performance. This paper also identifies which path is more important between the three behaviors through multi-mediation analysis. The results find that knowledge-sharing behavior better explains the relationship between psychological well-being and performance than the other two behaviors. This contributes to the research fields of psychological well-being and performance.

Second, although extensive research on the antecedents of knowledge-management has been conducted, there is a lack of research on the effects of psychological well-being on all categories of knowledge-management (Chumg et al., 2015). Psychological well-being is a manifestation of one’s strengths and a direct assessment of growth, directly affecting the attitudes and behaviors of members. Knowledge-sharing is a voluntary contribution to knowledge exchange in which psychological well-being must be taken into account because it greatly affects the internal motivation of members. This study contributes to the field of knowledge management by identifying the effects of psychological well-being on three dimensions of knowledge-management behavior.

Third, this paper contributes to the field of leadership research by identifying the impact of LMX as a contextual factor. Leaders should motivate subordinates to share knowledge. This study examined the influence of LMX to identify how different manifestations of leadership affect knowledge-management behavior. From the analysis, the better the relationship with the leader, the greater the participation in knowledge-sharing and the better the outcome. Therefore, if an organization strives to improve the performance of individuals through active knowledge-sharing and the performance of the organization, it is necessary to improve the quality of exchange relationships with members.

This paper has several practical implications. First, the dilemma of the knowledge-sharing situation experienced by the members of the organization was identified. Existing knowledge-management systems attempt to promote knowledge-sharing by providing intrinsic and extrinsic rewards. This paper finds that knowledge-sharing is not simply an action caused by the amount of a reward. Instead, knowledge-sharing is a conflict between the motive for expressing one’s ability by sharing and seeking recognition and compensation, and the desire to maintain exclusive rights by hiding and monopolizing knowledge to increase bargaining power and status. This paper has the same direction as the academic attempt to interpret knowledge sharing using game theory (Kong et al., 2020). Therefore, knowledge-management systems should be improved not only by providing compensation but also by eliminating the risk of sharing knowledge and securing the right to own knowledge. This is consistent with previous studies of knowledge ownership.

Second, this paper identified the process of the virtuous circle relationship between the happiness and performance of the members of the organization. According to the theory of positive organizational behavior, the happiness of members is the goal of organizations, but the continuous growth of the organization is essential to accomplish this. Knowledge-management is essential to organizational sustainability. This paper suggests a virtuous cycle between sustainable management and promoting the happiness of members by linking happiness-management to knowledge-management.

Third, LMX is the quality of exchange between leaders and members. As a result of this paper, if LMX plays a major role in knowledge sharing, it could play a big role not only in sharing knowledge among members but also in sharing knowledge between leaders and members. Knowledge sharing is possible in vertical as well as horizontal. Therefore, leadership training should be conducted with the core of the exchange relationship with the members (Dysvik et al., 2015).

### Limitations and Future Research

Despite these implications, this study has several limitations. First, various factors that could affect the relationship between psychological well-being and performance through knowledge-management behavior were not included in the research model. As explained earlier in the literature review, various factors influence knowledge-management behaviors, such as organizational culture or atmosphere, industry, policy, type of job, the existence of knowledge-management systems, and personality (Mustika et al., 2019). To analyze the relationship between psychological well-being, knowledge-management activities, and performance, the effects of these factors should be included or controlled. Second, this study’s context was Korean organizations and reflected the characteristics of Korean organizations. Korean organizations are collectivistic, and their members have relatively rigid relationships with leaders. The tendency to express psychological well-being outwardly is also conservative. In a culture where emotions are free to be expressed, the causality of the results of this study may be stronger. In the future, cross-validation should be carried out for multiple cultures. Third, this study tried to eliminate the common method bias by separating measurement time points and measuring performance from leaders, but psychological well-being and knowledge-management behaviors are self-reported and are not free from bias. If possible, future studies should use a comparison with indicators measured from peers or supervisors.

## Conclusion

The study findings contribute to the existing theory on organizational psychology, knowledge management, and leadership. The relationships between psychological well-being and three-types of knowledge management behavior were examined and the moderation effect of LMX on the relationship was verified. Also, knowledge management behavior-performance relationship was tested. The research findings imply that psychological well-being has a positive effect on the knowledge-sharing behavior, and negative effects on the knowledge hiding and manipulating behaviors. LMX enhances this positive effect. Also, knowledge-sharing behavior has a positive impact on individual performance while knowledge-hiding and manipulating behavior have a negative impact. Knowledge-sharing behavior significantly mediated the relationship between psychological well-being and performance. Psychological well-being has been empirically confirmed as the solution to the knowledge sharing dilemma. This study findings also suggested that LMX is an essential factor of solving knowledge sharing dilemmas. This study contributes to the field of positive psychology and presents empirical evidence that can be used to develop leadership and knowledge sharing motive.

## Data Availability Statement

The raw data supporting the conclusions of this article will be made available by the authors, without undue reservation.

## Ethics Statement

The studies involving human participants were reviewed and approved by Seoul National University. The patients/participants provided their written informed consent to participate in this study.

## Author Contributions

CK conceived, designed, and awarded the grant for the study. CK analyzed the data and wrote and discussed the manuscript.

## Conflict of Interest

The author declares that the research was conducted in the absence of any commercial or financial relationships that could be construed as a potential conflict of interest.

## References

[B1] Bandura A. (1997). *Self-Efficacy: The Exercise of Control.* New York NY: Freeman, 10.1891/0889-8391.13.2.158

[B2] Bernerth J. B. Taylor S. G. Walker H. J. Whitman D. S. (2012). An empirical investigation of dispositional antecedents and performance-related outcomes of credit scores. *J. Appl. Psychol.* 97 469–478. 10.1037/a0026055 22023073

[B3] Bettis-Outland H. (1999). The impact of information distortion within the context of implementing and sustaining a market orientation. *J. Strateg. Mark.* 7 251–263. 10.1080/096525499346369

[B4] Blau P. M. (1964). *Exchange and Power in Social Life.* New York NY: John Wiley & Sons, Inc, 10.2307/2574842

[B5] Bock G.-W. Kim Y.-G. (2002). Breaking the myths of rewards: an exploratory study of attitudes about knowledge sharing. *Inf. Resour. Manag.* 15 14–21. 10.4018/irmj.2002040102

[B6] Bock G.-W. Zmud R. W. Kim Y.-G. Lee J.-N. (2005). Behavioral intention formation in knowledge sharing: examining the roles of extrinsic motivators, social-psychological forces, and organizational climate. *MIS Q.* 29 87–111. 10.2307/25148669

[B7] Brayfield A. H. Crockett W. H. (1955). Employee attitudes and employee performance. *Psychol. Bull.* 52 396–424. 10.1037/h0045899 13266929

[B8] Brown J. S. Duguid P. (1991). Organizational learning and communities-of-practice: toward a unified view of working, learning, and innovation. *Organ. Sci.* 2 40–57. 10.1287/orsc.2.1.40 19642375

[B9] Bryant S. E. (2003). The role of transformational and transactional leadership in creating, sharing and exploiting organizational knowledge. *J. Leadersh. Organ. Stud.* 9 32–44. 10.1177/107179190300900403

[B10] Cabrera A. Collins W. C. Salgado J. F. (2006). Determinants of individual engagement in knowledge sharing. *J. Hum. Resour. Manag.* 17 245–264. 10.1080/09585190500404614

[B11] Carolan S. Harris P. R. Cavanagh K. (2017). Improving employee well-being and effectiveness: systematic review and meta-analysis of web-based psychological interventions delivered in the workplace. *J. Med. Internet. Res.* 19:e271. 10.2196/jmir.7583 28747293 PMC5550734

[B12] Chaurasia S. Shukla A. (2013). The influence of leader-member exchange relations on employee engagement and work role performance. *Int. J. Organ. Theory Behav.* 16 465–493. 10.1108/ijotb-16-04-2013-b002

[B13] Chiu C. M. Hsu M. H. Wang E. T. G. (2006). Understanding knowledge sharing in virtual communities: an integration of social capital and social cognitive theories. *Decis. Support Syst.* 42 1872–1888. 10.1016/j.dss.2006.04.001

[B14] Chumg H. F. Cooke L. Fry J. Hung I. H. (2015). Factors affecting knowledge sharing in the virtual organisation: Employees’ sense of well-being as a mediating effect. *Comput. Hum. Behav.* 44 70–80. 10.1016/j.chb.2014.11.040

[B15] Collins C. J. Smith K. G. (2006). Knowledge exchange and combination: the role of human resource practices in the performance of high-technology firms. *Acad. Manage. J.* 49 544–560. 10.5465/amj.2006.21794671

[B16] Connelly C. E. Zweig D. Webster J. Trougakos J. P. (2012). Knowledge hiding in organizations. *J. Organ. Behav.* 33 64–88. 10.1002/job.737

[B17] Constant D. Kiesler S. Sproull L. (1994). What’s mine is ours, or is it? A study of attitudes about information sharing. *Inf. Syst. Res.* 5 400–421. 10.1287/isre.5.4.400 19642375

[B18] Cropanzano R. Mitchell M. S. (2005). Social exchange theory: an interdisciplinary review. *J. Manage.* 31 874–900. 10.1177/0149206305279602

[B19] Cummings J. N. (2004). Work groups, structural diversity, and knowledge sharing in a global organization. *Manage. Sci.* 50 352–364. 10.1287/mnsc.1030.0134 19642375

[B20] Dansereau F. Graen G. Haga W. J. (1975). A vertical dyad linkage approach to leadership within formal organizations: a longitudinal investigation of the role making process. *Organ. Behav. Hum. Perform.* 13 46–78. 10.1016/0030-5073(75)90005-7

[B21] Darroch J. (2005). Knowledge management, innovation and firm performance. *J. Knowl. Manag.* 9 101–115. 10.1108/13673270510602809

[B22] Dysvik A. Buch R. Kuvaas B. (2015). Knowledge donating and knowledge collecting: the moderating roles of social and economic LMX. *Leadersh. Organ. Dev. J.* 36 35–53. 10.1108/lodj-11-2012-0145

[B23] Eagly A. H. Chaiken S. (1993). *The Psychology of Attitudes.* Fort Worth, TX: Harcourt Brace Jovanovich, 10.1002/mar.4220120509

[B24] Garg P. Rastogi R. (2009). Effect of psychological wellbeing on organizational commitment of employees. *Icfai Univ. J. Organ. Behav.* 8 42–51. 10.15373/2249555x/feb2013/70

[B25] Graen G. B. Uhl-Bien M. (1995). Relationship-based approach to leadership: Development of leader-member exchange (LMX) theory of leadership over 25 years: Applying a multi-level multi-domain perspective. *Leadersh. Q.* 6 219–247. 10.1016/1048-9843(95)90036-5

[B26] Grant R. M. (2002). “The knowledge-based view of the firm,” in *The Strategic Management of Intellectual Capital and Organizational Knowledge*, eds Choo C. W. Bontis N. (Oxford: Oxford University Press), 133–148. 10.1093/oxfordhb/9780199275212.003.0008

[B27] Hu L. T. Bentler P. M. (1999). Cutoff criteria for fit indexes in covariance structure analysis: conventional criteria versus new alternatives. *Struct. Equ. Modeling.* 6 1–55. 10.1080/10705519909540118

[B28] Jarvenpaa S. L. Staples D. S. (2000). The use of collaborative electronic media for information sharing: an exploratory study of determinants. *J. Strateg. Inf. Syst.* 9 129–154. 10.1016/s0963-8687(00)00042-1

[B29] Jena L. K. Pradhan S. Panigrahy N. P. (2018). Pursuit of organisational trust: role of employee engagement, psychological well-being and transformational leadership. *Asia Pacific Manag. Rev.* 23 227–234. 10.1016/j.apmrv.2017.11.001

[B30] Joo B. K. B. Park J. G. Lim T. (2016). Structural determinants of psychological well-being for knowledge workers in South Korea. *Pers. Rev.* 45 1069–1086. 10.1108/PR-01-2015-0011

[B31] Kankanhalli A. Tan B. C. Y. Wei K.-K. (2005). Contributing knowledge to electronic knowledge repositories: an empirical investigation. *MIS Q.* 29 113–143. 10.2307/25148670

[B32] Keaveney S. M. Nelson J. E. (1993). Coping with organizational role stress: intrinsic motivational orientation, perceived role benefits, and psychological withdrawal. *J. Acad. Mark. Sci.* 21 113–124. 10.1007/BF02894422

[B33] Kim S. Lee H. (2006). The impact of organizational context and information technology on employee knowledge-sharing capabilities. *Public Adm. Rev.* 66 370–385. 10.1111/j.1540-6210.2006.00595.x

[B34] Kimmerle J. Wodzicki K. Jarodzka H. Cress U. (2011). Value of information, behavioral guidelines, and social value orientation in an information-exchange dilemma. *Group Dyn.* 15 173–186. 10.1037/a0021467

[B35] Kong X. Xu Q. Zhu T. (2020). Dynamic evolution of knowledge sharing behavior among enterprises in the cluster innovation network based on evolutionary game theory. *Sustainability* 12 75–97. 10.3390/su12010075

[B36] Kwok S. H. Gao S. (2005). Attitude towards knowledge sharing behavior. *J. Comput. Inf. Syst.* 46 45–51. 10.14711/thesis-b834876

[B37] Lee J.-H. Kim Y.-G. Kim M.-Y. (2006). Effects of managerial drivers and climate maturity on knowledge-management performance: empirical validation. *Inf. Resour. Manag. J.* 19 48–60. 10.4018/irmj.2006070104

[B38] Lin H. F. (2007). Knowledge sharing and firm innovation capability: an empirical study. *Int. J. Manpow.* 28 315–332. 10.1108/01437720710755272

[B39] Liden R. C. Graen G. (1980). Generalizability of the vertical dyad linkage model of leadership. *Acad. Manage. J.* 23 451–465. 10.5465/255511

[B40] Luthans F. Luthans K. W. Luthans B. C. (2004). Positive psychological capital: beyond human and social capital. *Bus. Horiz.* 47 45–50. 10.1016/j.bushor.2003.11.007

[B41] Mustika H. Eliyana A. Agustina T. S. (2019). Antecedents of knowledge sharing behaviour. *Int. J. Innov. Creativity Chang.* 8 57–71. 10.9770/jssi.2020.9.m(12)

[B42] Ohlsson S. (2011). *Deep Learning: How the Mind Overrides Experience.* Cambridge: Cambridge University Press, 10.1017/CBO9780511780295

[B43] Ordieres-Meré J. Remón T. P. Rubio J. (2020). Digitalization: an opportunity for contributing to sustainability from knowledge creation. *Sustainability* 12 1460–1481. 10.3390/su12041460

[B44] Preacher K. J. Hayes A. F. (2004). SPSS and SAS procedures for estimating indirect effects in simple mediation models. *Behav. Res Methods Instrum. Comput.* 36 717–731. 10.3758/bf03206553 15641418

[B45] Rhee Y. W. Choi J. N. (2017). Knowledge management behavior and individual creativity: goal orientations as antecedents and in-group social status as moderating contingency. *J. Organ. Behav.* 38 813–832. 10.1002/job.2168

[B46] Roethlisberger F. J. Dickson W. J. (1939). *Management and the Worker.* Cambridge. MA: Harvard University Press, 10.1177/000271624020800170

[B47] Ryff C. D. (1989). Happiness is everything, or is it? Explorations on the meaning of psychological well-being. *J. Pers. Soc. Psychol.* 57 1069–1081. 10.1037/0022-3514.57.6.1069

[B48] Ryff C. D. Keyes C. L. M. (1995). The structure of psychological well-being revisited. *J. Pers. Soc. Psychol.* 69 719–727. 10.1037/0022-3514.69.4.719 7473027

[B49] Shore L. M. Martin H. J. (1989). Job satisfaction and organizational commitment in relation to work performance and turnover intentions. *Hum. Relat.* 42 625–638. 10.1177/001872678904200705

[B50] Srivastava A. Bartol K. M. Locke E. A. (2006). Empowering leadership in management teams: effects on knowledge sharing, efficacy, and performance. *Acad. Manage. J.* 49 1239–1251. 10.5465/amj.2006.23478718

[B51] Taylor W. A. Wright G. H. (2004). Organizational readiness for successful knowledge sharing: challenges for public sector managers. *Inf. Resour. Manag. J.* 17 22–37. 10.4018/irmj.2004040102

[B52] Wang S. Noe R. A. (2010). Knowledge sharing: a review and directions for future research. *Hum. Resour. Manag. Rev.* 20 115–131. 10.1016/j.hrmr.2009.10.001

[B53] Wang Y. Han M. S. Xiang D. Hampson D. P. (2019). The double-edged effects of perceived knowledge hiding: empirical evidence from the sales context. *J. Knowl. Manag.* 23 279–296. 10.1108/jkm-04-2018-0245

[B54] Williams L. J. Anderson S. E. (1991). Job satisfaction and organizational commitment as predictors of organizational citizenship and in-role behaviors. *J. Manage.* 17 601–617. 10.1177/014920639101700305

[B55] Yao L. J. Kam T. H. Y. Chan S. H. (2007). Knowledge sharing in Asian public administration sector: the case of Hong Kong. *J. Enterp. Inf. Manag.* 20 51–69. 10.1108/17410390710717138

[B56] Yukl G. (1989). Managerial leadership: a review of theory and research. *J. Manage.* 15 251–289. 10.1177/014920638901500207

